# Supplementary material for: Cryo-EM Map Anisotropy Can Be Attenuated by Map Post-Processing and a New Method for Its Estimation
Source: Int J Mol Sci. 2024 Apr 2;25(7):3959. doi: 10.3390/ijms25073959 (PMC11012471; doi:10.3390/ijms25073959)
Supplement: Supplementary file 1 [file ijms-25-03959-s001.zip › ijms-2907499-supplementary.pdf]

## Supporting information

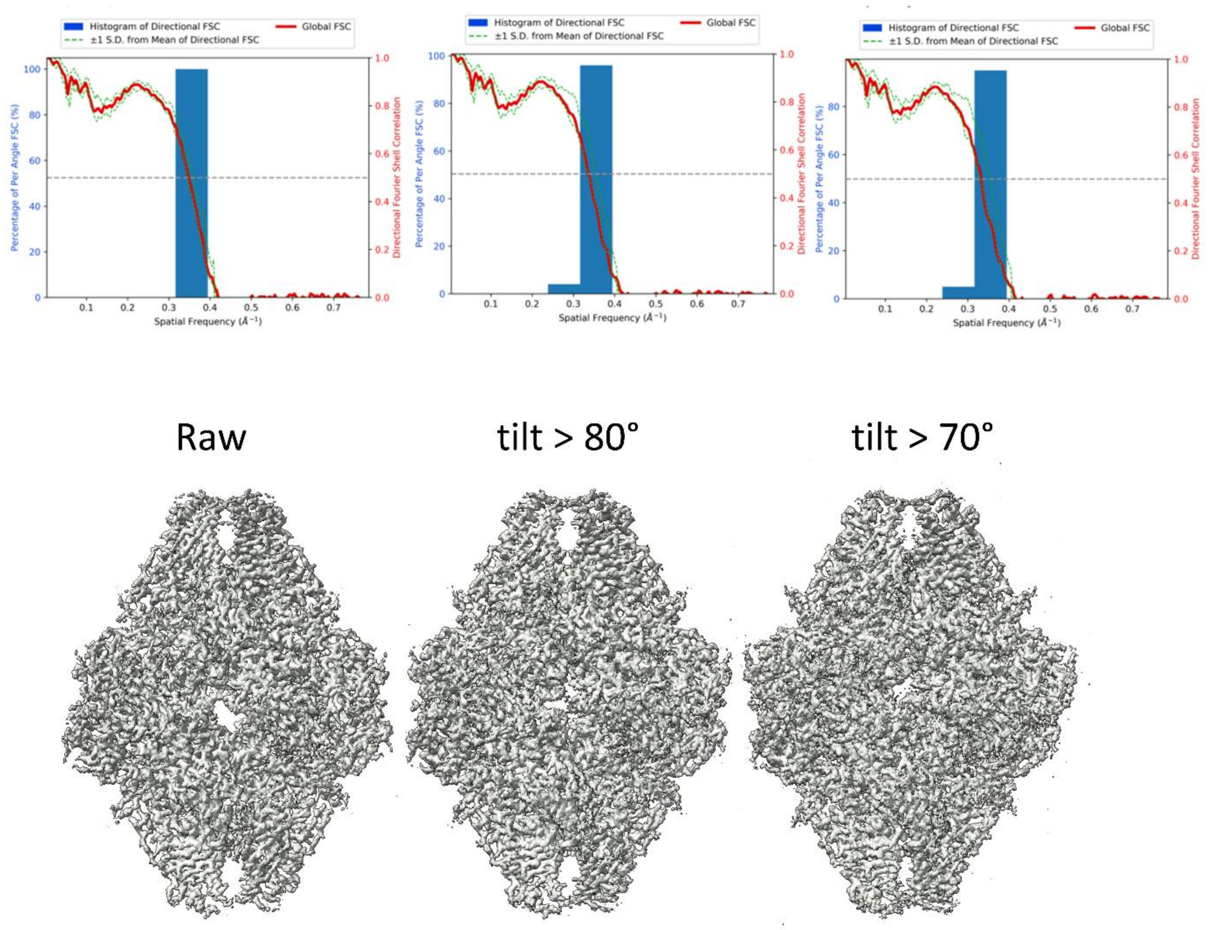


**Figure S1.** Bottom, cryo-EM maps for the β -galactosidase reconstructed from the EMPIAR-10061 dataset, using particles from all the projection directions (Left), and removing 95% of the particles with tilt angle >80° (Middle) and >70° (Right). Top, 3D-FSC displaying the global FSC resolution (red) and standard deviation of the directional resolution over 20-degree cones (green) as well as the histogram of the per angle FSC for the raw β-galactosidase map (Left), and the maps reconstructed with 95% of the particles with tilt angle >80° (Middle) and >70° (Right) removed.

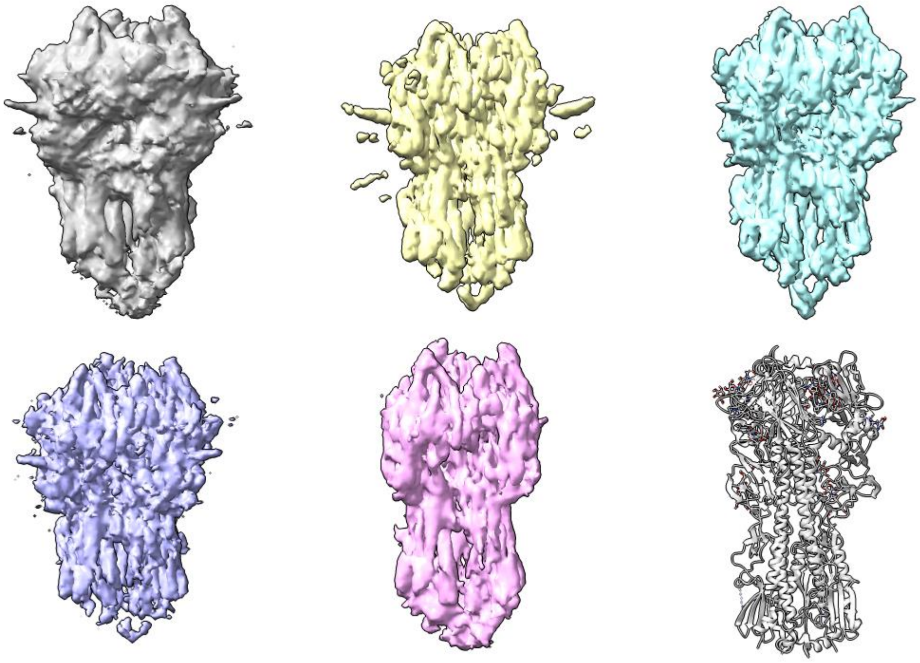

**Figure S2.** Hemagglutinin trimer map reconstructed from untilted micrographs (grey), and the results of applying DeepEMhancer (yellow, [21]), LocSpiral (cyan, [24]), LocalDeblur (purple, [25]), and LocScale2 (pink, [22,23]) to the reconstructed map. The atomic model used for evaluation is depicted in pale grey.

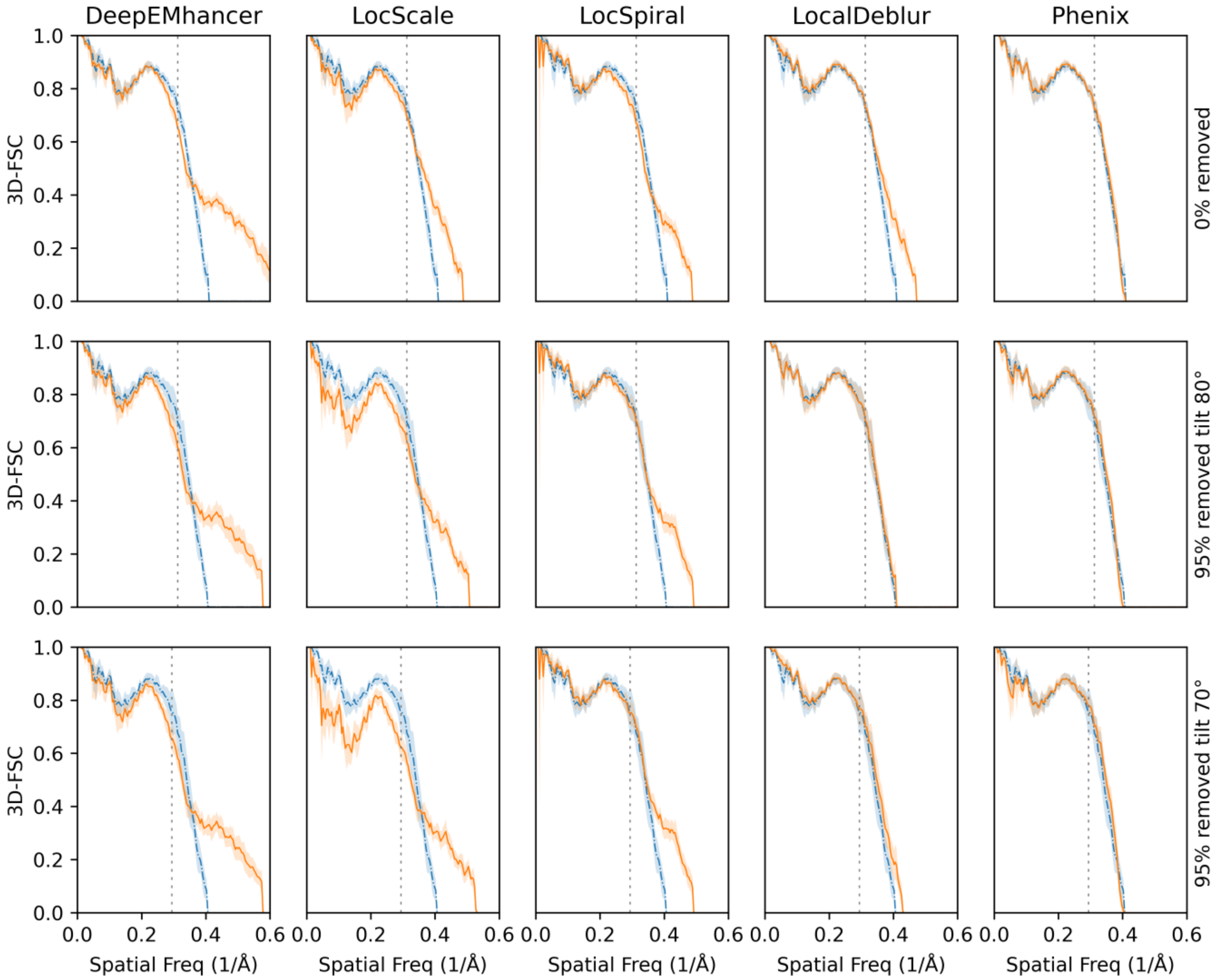


**Figure S3.** Alternative representation of the per-cone map to model FSC (mean, solid lines, standard deviation, shadow) calculated with 3D-FSC for three different versions of the β-galactosidase complex: reconstructed with all particles (Top) and reconstructed with 95% of the particles with tilt angle >80° (Middle) and >70° (Bottom) removed. The blue line represents the reconstructed map whereas the orange lines represent the maps obtained when DeepEMhancer [21], LocScale2 [22,23], LocSpiral [24], Phenix [15] and LocalDeblur [25] are employed on the reconstructed map. The FSC global resolution is displayed as a dotted vertical line.

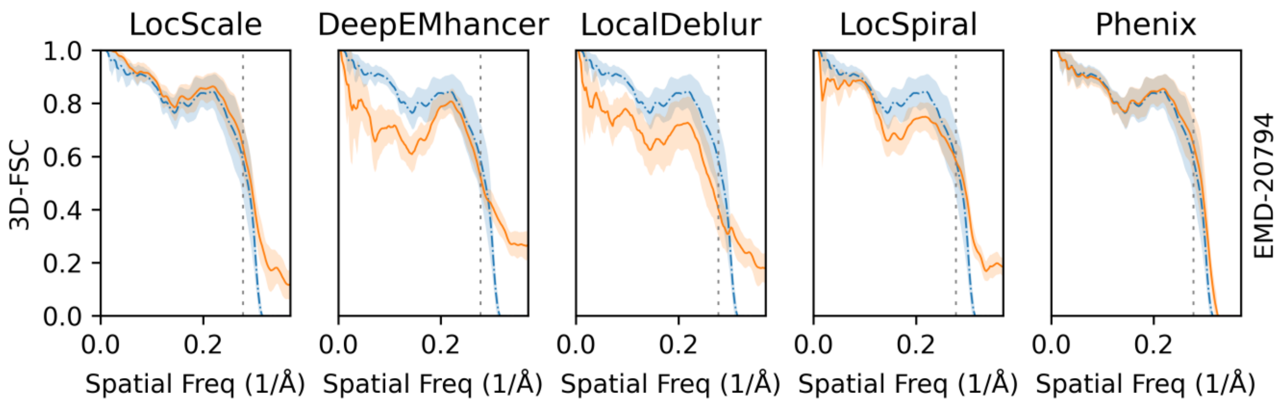


**Figure S4.** Alternative representation of the per-cone map to model FSC (mean, solid lines, standard deviation, shadow) for the EMD-20794. The blue line represents the reconstructed map whereas the orange lines represent the standard deviation for the maps obtained when DeepEMhancer [21], LocScale2 [22,23], LocSpiral [24], Phenix [15] and LocalDeblur [25] are employed on the reconstructed map. The FSC global resolution is displayed as a dotted vertical line.

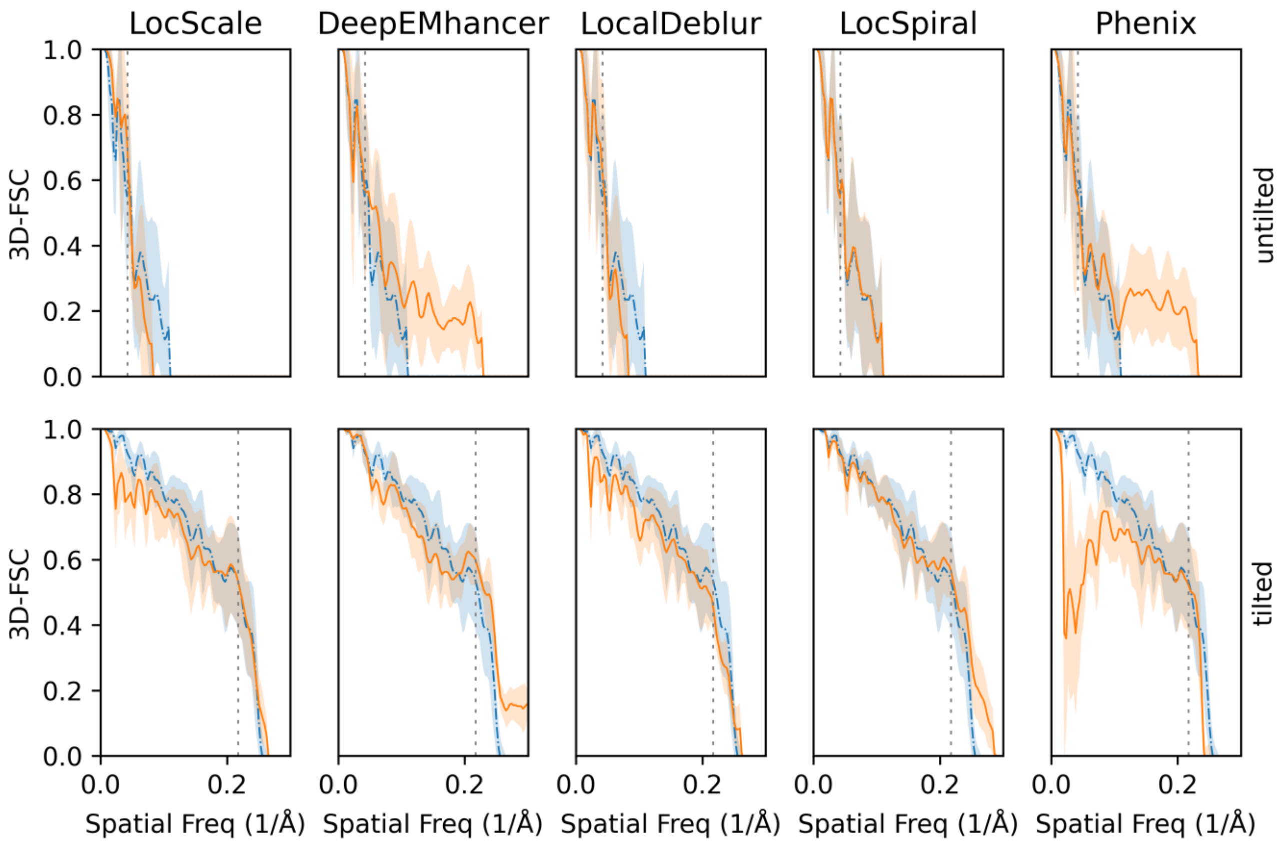


**Figure S5.** Alternative representation of the per-cone map to model FSC (mean, solid lines, standard deviation, shadow) calculated with 3D-FSC for two different versions of the influenza hemagglutinin trimer: reconstructed with untilted micrographs (severe anisotropy problems) and reconstructed with micrographs tilted 40°. The blue line represents the reconstructed map whereas the orange lines represent the maps obtained when DeepEMhancer [21], LocScale2 [22,23], LocSpiral [24], Phenix [15] and LocalDeblur [25] are employed on the reconstructed map. The FSC global resolution is displayed as a dotted vertical line.

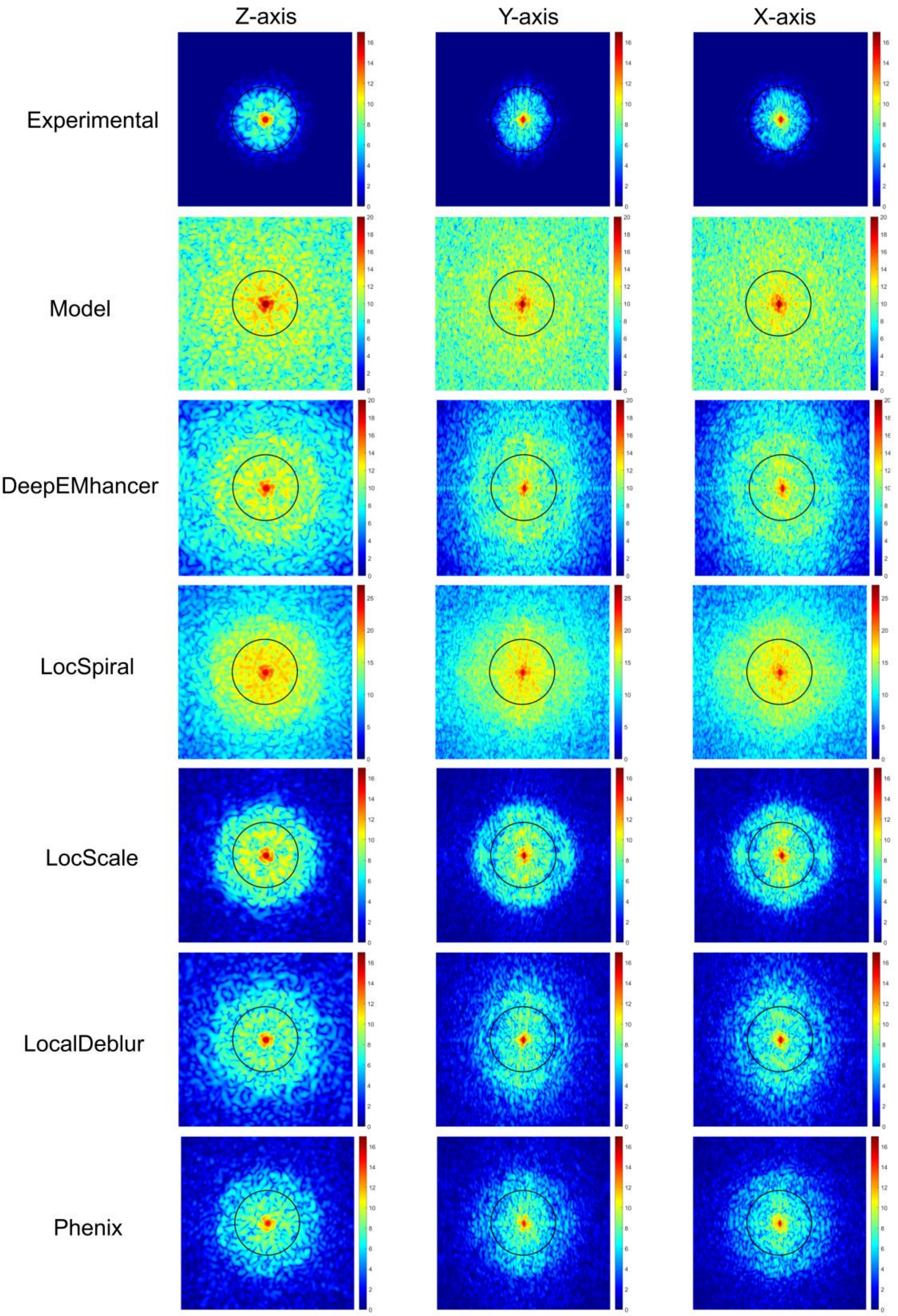


**Figure S6.** Logarithm of Fourier amplitudes for the tilted influenza hemagglutinin trimer reconstructions at slices $z = 0$, $y = 0$ and $x = 0$ after running the different postprocessing approaches. The first and second rows correspond to the experimental map without any postprocessing (first row) and the map simulated from the atomic model (second row). The black circle in the figures shows a resolution corresponding to 3.5 Å.

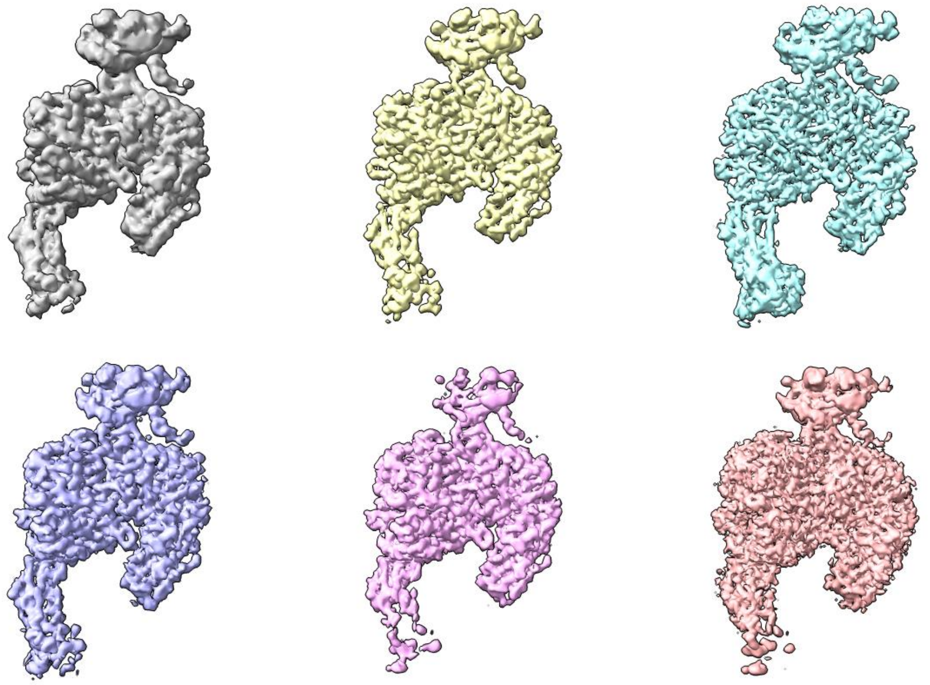

**Figure S7.** Reconstructed map (grey) for the EMD-20794 and the results of applying DeepEMhancer (yellow, [21]), LocSpiral (cyan, [24]), LocalDeblur (purple, [25]), LocScale2 (pink, [22,23]) and Phenix anisotropic sharpening (salmon, [15]) to the reconstructed map.

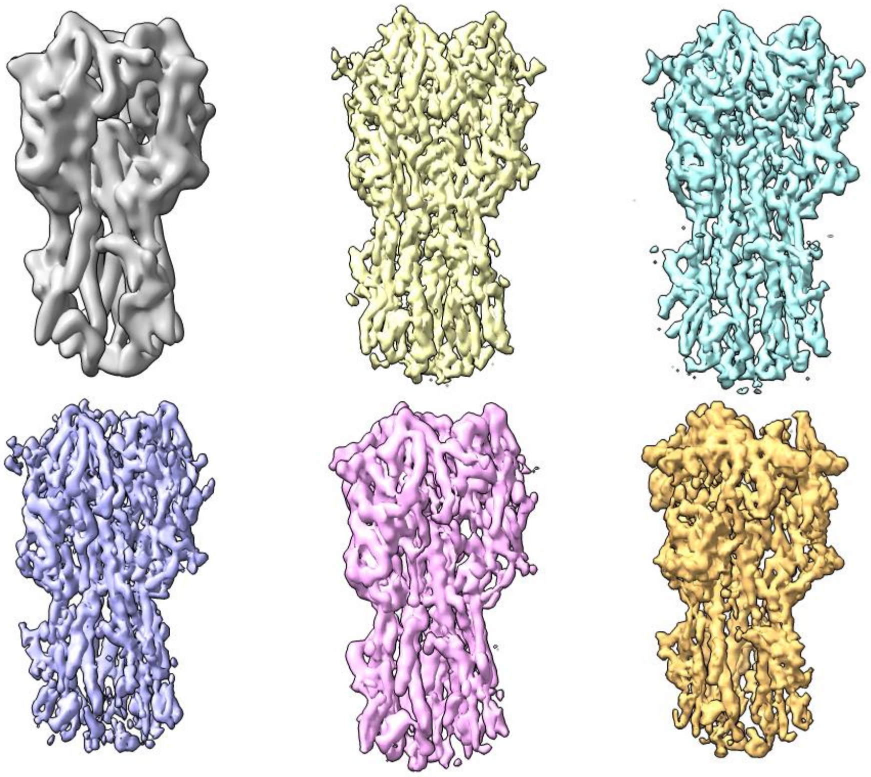

**Figure S8.** Influenza hemagglutinin trimer map reconstructed from tilted micrographs (grey), and the results of applying DeepEMhancer (yellow, [21]), LocSpiral (cyan, [24]), LocalDeblur (purple, [25]), LocScale2 (pink, [22,23]) and Phenix anisotropic sharpening (salmon, [15]) to the reconstructed map. The atomic model used for evaluation is depicted in pale grey.
